# Durability and Aesthetics of Architectural Concrete under Chloride Attack or Carbonation

**DOI:** 10.3390/ma13040839

**Published:** 2020-02-12

**Authors:** Honglei Chang, Penggang Wang, Zuquan Jin, Gang Li, Pan Feng, Shoujie Ye, Jian Liu

**Affiliations:** 1 School of Qilu Transportation, Shandong University, Jinan 250002, China; hlchang@sdu.edu.cn (H.C.); lj75@sdu.edu.cn (J.L.); 2 School of Civil Engineering, Qingdao University of Technology, Qingdao 266033, China; 3 Cooperative Innovation Center of Engineering Construction and Safety in Shandong Blue Economic Zone, Qingdao 266033, China; 4 Qingdao Metro Group Co., Ltd., Qingdao 266000, China; cementitious_3d@163.com (G.L.); yeshoujie813@163.com (S.Y.); 5 School of Material Science and Engineering, Southeast University, Nanjing 211189, China; pan.feng@seu.edu.cn

**Keywords:** architectural concrete, marine environment, chloride penetration, carbonation, surface appearance

## Abstract

Architectural concrete has been wildly used nowadays, and those served in an offshore environment often suffer from chloride penetration and carbonation. To assess the protection and decoration performances of architectural concrete, this study exposed architectural concrete to actual marine environments and accelerated carbonation conditions. The chloride and carbonation resistance of architectural concrete was determined to evaluate the protection performance, and the corresponding surface-color-consistency was adopted to characterize its decoration performance. The results show that the total and free chloride of concrete in the marine atmosphere zone and the tidal zone generally decreases with depth; chloride content arguments significantly with exposure time, with a chloride maximum peak near the surface. Moreover, the chloride diffusion coefficient is small throughout the measurements, indicating the superior chloride resistance of architectural concrete. Furthermore, architectural concrete also possesses excellent carbonation resistance based on the carbonation depth data obtained from the carbonation experiment. Therefore, architecture concrete served as protection covers can withstand both the chloride attack and carbonation tested in this paper. In addition, carbonation was found to have a profound influence on the aesthetics of architectural concrete. Therefore, carbonation should be carefully handled for better maintaining the aesthetic appearance of architectural concrete in long-term service.

## 1. Introduction

Architectural concrete is one way of modernist architecture expression, also christened decoration concrete for its valuable decorative effect. Architectural concrete, being environment-friendly, economic, and aesthetical, has been widely used in construction projects recently [1,2]. For instance, more and more art galleries, cultural corridors, stadiums, and high-speed rail stations in China have incorporated architectural concrete.

Without the protection of exterior decoration materials, architectural concrete is prone to be influenced by different factors during service. Indoors, it is subjected to carbonation effect; outdoor, it may be vandalized by wind, lower temperature, and cyclic wetting–drying; in coastal areas, it may suffer from chloride attack. All those adversities mentioned above will not only threaten the durability of architectural concrete structures but damage its shiny surface and defile its beauty. 

**Figure 1:**
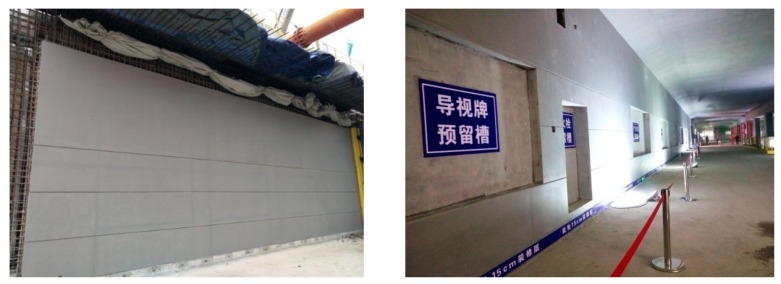
Architectural concrete applied in Qingdao subway station.

Many cities in China are now constructing subways, for example Qingdao City. Metro Line 3 in Qingdao has employed architectural concrete for decoration (see Figure 1). Considering that Qingdao is a sea city, corrosive ions such as chlorides in the sea can be transported into the subway (especially the offshore ones) carried by wind and fog, which will not only accelerate the corrosion of steels in concrete but may damage the appearance of architectural concrete. Moreover, the high CO_2_ concentration caused by the dense population in the subway probably makes the situation even worse. 

The chloride penetration resistance and carbonation resistance of concrete have been widely investigated [3,4,5,6,7,8,9,10], which are not needed to be stated extensively. So far, the studies of architectural concrete mainly focus on construction techniques [11,12,13,14,15]. Li [11] and Liu [12] reviewed the development, problems, and countermeasures of architectural concrete, and pointed out its promising applications. Huangong [13] and Wang [14] introduced the construction techniques of architectural concrete and proposed methods for improving construction quality. Using Tianjing Museum as the application background, Chen [15] elaborated a complete set of construction technology of architectural concrete from material selection, mix design, stirring technology, template selection, vibrating method, and curing. Moreover, Liu and Li [16,17] designed and optimized the mix proportions of architectural concrete, evaluated the mechanical property and durability, and successfully applied it in the construction of Metro Line 3 in Qingdao. Lv [18] explored the durability of architectural concrete based on experiments and practical engineering, finding that several mix proportions all possessed superior durability. Catia [19] presented an expert system to support the inspection, diagnosis, and repair of architectural concrete surfaces, and aims to improve the quality of architectural concrete. Besides, to improve the modern plasticity in youth buildings, Lim [20] probed into the application and property of precast architectural concrete panels. However, studies about the durability and surface appearance evaluation of architectural concrete in marine environments, which helps the acquiring of safety indicators and the controlling of harmful factors, have rarely been reported, even though the chloride penetration resistance and carbonation resistance of concrete has been widely investigated [3,4,5,6,7,8,9,10].

Therefore, this study prepared architectural concrete specimens with mix proportions employed in the subway construction of Qingdao city, and exposed them to the actual marine environments to evaluate their chloride penetration resistance and explore the impact of chloride attack on their surface appearance in the chloride attack atmosphere conditions. Furthermore, the anti-carbonation property and the influence of carbonation on surface appearance were also investigated through accelerated carbonation experiments.

## 2. Experiments

### 2.1. Raw Materials and Mix Proportions

In this research, Portland Cement P.I. 52.5 was used, and its chemical composition is listed in Table 1. Grade I fly ash (FA) and Grade S95 granulated blast-furnace slag (SL) were employed as mineral admixtures, and their chemical compositions are also given in Table 1. Fine aggregates are medium river sand with fineness modulus of 2.6, and coarse aggregates are continuous grading gravels with a diameter of 5–25 mm. In addition, polycarboxylate superplasticizer (SP) was adopted to reduce water. 

Three strength grades of architectural concrete were adopted in this study, named C40, C50, and C55 respectively. And the three mix proportions are presented in Table 2. 

### 2.2. Sample Preparation 

Architectural concrete has a high demand for surface quality. Therefore, molds need to be cleaned up and covered with special release agent inside before casting. After blending and stirring, the slump and dispersion degree of concrete was first tested [21]. Detailedly, fresh concrete was filled in a horn slump cone of 100 mm top mouth diameter, 200 mm bottom diameter, and 300 mm height. Note that concrete-filling was operated in steps, accompanied by constant stirring with a steel bar. After filling, the slump cone was lifted up, and the altitude difference between the slump cone height and the highest point of the slumped concrete was tested, that is, the slump of the fresh concrete. Then, the maximum and minimum diameter of the slumped concrete was tested and the average value calculated was deemed the extended degree of fresh concrete. Then, the rest was filled into molds of different sizes three times, 1/3 each time until full and vibrating after each filling.

Cubic specimens of size 100 mm × 100 mm × 100 mm were used for compressive strength test, chloride penetration resistance test, and carbonation resistance test. They were demolded after casting for 24 h and then placed in the curing room of 20 ± 1 °C temperature and ≥95% relative humidity. The specimens for compressive strength were cured for 3 days, 28 days, and 56 days, separately. And the other specimens were cured for 28 days.

**Figure 2:**
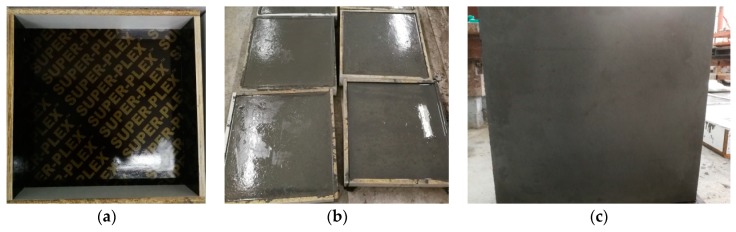
Casting process of specimens for surface appearance test. (**a**) Wooden mold. (**b**) Specimens after casting. (**c**) Specimens after demolding.

Specimens for surface appearance tests were molded in special wooden molds of size 500 mm × 500 mm × 100 mm. They were demolded after casting for 48 h and cured in the curing room for 28 days. Then, they were cut into four equal-sized pieces of size 250 mm × 250 mm × 100 mm for the following surface appearance test. The mold specimens after casting and demolding are shown in Figure 2.

### 2.3. Compressive Strength

The compressive strength of concrete was tested abiding by the standard for the test method of mechanical properties on ordinary concrete [22]. After reaching the curing age, the compressive strength of specimens was tested using a 300 t compressive strength tester (Shijin, Jinan, China) with a loading of 0.5 MPa/s. Three specimens of each mix were randomly chosen, and the average value of them was used for analysis.

### 2.4. Carbonation

Specimens cured for 28 days were dried in the 60 °C drying oven (Baihui, Guangzhou, China) for 48 h, Then, except 2 opposites, the other 4 surfaces of specimens were sealed with epoxy resin, and then put into a carbonation tank. The carbonation time was 14 days, 28 days, and 56 days separately. The temperature in the carbonation tank was 20 ± 1 °C, humidity 75% ± 2%, and carbonation concentration 20% ± 1% [23]. After reaching the carbonation time, specimens were split and sprayed with alcohol phenolphthalein solution to test carbonation depth. The ultimate carbonation depth is the average of the 3 specimens of each age.

Note that the accelerated carbonation method instead of the natural exposure method was employed to evaluate the carbonation resistance property of architectural concrete. The accelerated carbonation method has been widely used in many studies [24,25,26,27,28] in view of its efficiency. Besides, this method is also quite feasible as has been demonstrated by reported investigations [24,25,29,30,31]. Moreover, the accelerated carbonation method has been written in a test standard [23] in China, which also illustrates the feasibility of this method.

### 2.5. Salt Fog Exposure

Salt fog exposure is to simulate the atmosphere zone of oceans that fosters chloride attack, which was combined with the experiments conducted in the real oceanic atmosphere, and in such a way, the influence of chloride attack on the surface appearance of architectural concrete was investigated. The corrosive solution is a NaCl solution of 5% by mass, and the spraying time is 12 h each day. After each spraying, specimens were taken out of the salt fog tank and dried in the atmospheric environment of constant temperature and humidity (20 ± 1 °C, 75% ± 2%) for 12 h, and then were sprayed with salt fog again [32]. That cycle was repeated during each exposure time: 14 days, 28 days, and 56 days, separately.

### 2.6. Marine Environments Exposure

**Figure 3:**
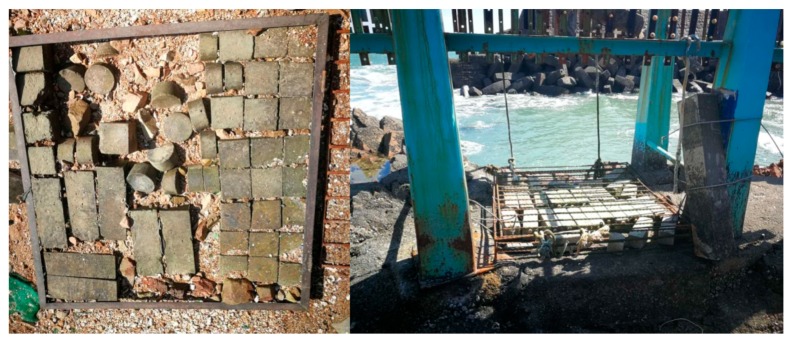
Architectural concrete exposed to actual marine environments.

After being cured for 28 days, five surfaces of each specimen were sealed with epoxy resin, and the remaining one was the exposure surface. Then, specimens were exposed to the atmosphere zone, tidal zone, and splash zone of Wheat Island marine exposure field in Qingdao (see Figure 3) for 1 month, 3 months, and 9 months, separately.

Specimens were ready to be tested after exposure. The powder samples for testing chloride content were obtained through grinding the specimen layer by layer. The grinding thickness of each layer was 1.0 mm in the range of 0–10 mm from the exposed surface, and 2.0 mm beyond a depth of 10 mm. Then, a silver nitration titration method was employed to test free chloride and total chloride in compliance with the Testing Code JTJ 270–98 [33]. The detailed operation can be found in reference [5,34]. In addition, note that the “free” and “total” chloride mentioned in this research is also referred to as water-soluble and acid-soluble chloride somewhere else, respectively.

### 2.7. Surface Appearance

In this research, the standard deviation of image gray was introduced to quantitatively characterize the chromatic change of faired-faced concrete surfaces. The standard deviation of image gray reflects the physical quantity of color difference of images, which is obtained through calculating the gray degree of gathered pixels and average gray degree of images [35], as shown in Equation (1).

(1) $$Std=\sqrt{\frac{\sum_{i=1}^{M}\sum_{j=1}^{N}{\left(Gray\left(i,j\right)-\bar{Gray}\right)}^{2}}{M\times N}}$$

where *Std* is standard deviation of image gray; *M × N* is the two-dimensional matrix, representing the total rows and columns; *Gray*(*i*,*j*) is the gray degree of the gathered pixels in images; $\bar{Gray}$ is the average gray degree of images.

The steps of obtaining the standard derivation of image gray are as follows: gather the surface image of architectural concrete with Canon EOS800D (8 million pixels) digital camera (Suning, Nanjing, China); covert the original format of the image into the gray image; calculate the average gray degree with the image processing software Image-Pro Plus (software version6.0, MEDIA CYBERNETICS, Rockville, MD, USA). The smaller the standard derivation of image gray is, the smaller the color difference of the image, and the more uniform the surface color [36]. To exclude the influence of environmental factors, the image gathering of architectural concrete with different mix proportions were conducted under the same indoor light condition, with the vertical distance between the digital camera and specimen surface being 1 m.

After preparing specimens of size 250 mm × 250 mm × 100 mm as mentioned in Section 2.2, the standard derivation of image gray of them was obtained following the steps above. Then, specimens were exposed to salt fog conditions, actual oceanic atmosphere, and carbonation conditions, separately, following the operation described above. After exposure, the standard derivation of architectural concrete specimens was obtained again for later discussion.

### 2.8. Capillary Absorption

Cubic specimens were cured for 28 days, and then were dried at constant temperature (20 ± 0.5 °C) and humidity (50% ± 10%) room for 7 days [32]. Then, they were placed in a plastic chamber contacting with 5% NaCl solution (by mass). The water level was kept approximately 3 mm above the contact surface of specimens. The absorbed mass of solution was determined as a function of time by weighing the specimens after the contact time of 0.5 h, 1 h, 2 h, 4 h, 8 h, 12 h, and 24 h. The capillary absorption coefficient of concretes was decided by Equation (2).

(2) $$\Delta W=A\cdot \Delta \sqrt{t}$$

where *ΔW* is the absorbed solution amount per unit cross-sectional area within time *t*, g/m^2^; *t* is absorbed time, h; *A* is the capillary absorption coefficient, g/(m^2^·h^0.5^).

### 2.9. Mercury Intrusion Porosimetry (MIP)

Specimens of size 100 mm × 100 mm × 100 mm were cast following Table 2. After curing for 28 days, several sheet pieces with a diameter of less than 2 mm were obtained from the inner part of specimens. Afterward, those samples were stored in ethyl alcohol for 7 days and then dried at 45 °C in a vacuum oven for another 7 days. Then, MIP was carried out by using equipment DV 2000 Micromeritics, Shanghai, China).

## 3. Results and Discussion

### 3.1. Basic Properties

The slump, extended degree, and compressive strength of architectural concrete are listed in Table 3. It can be seen that the slump and extended degree are large, which guarantees the feasibility of casting and helps to improve the quality of architectural concrete. In addition, it can be found that the strength of concrete of all mix proportions at 3 days has reached 70%–80% of designed strength, and the strength of 28 days has already met the design value.

### 3.2. Capillary Absorption Capacity

**Figure 4:**
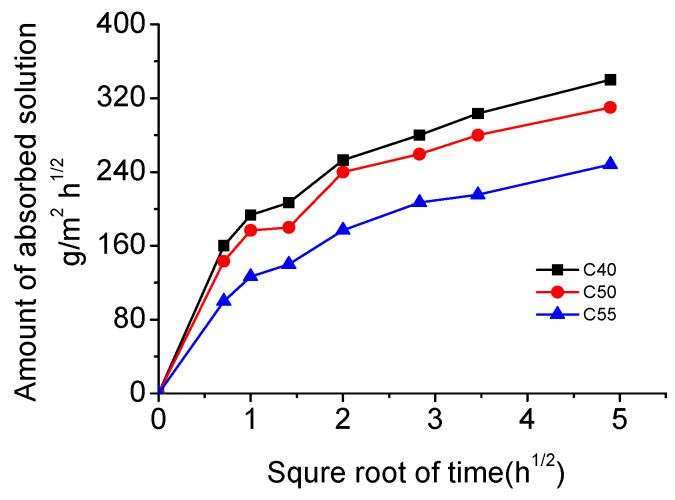
Amount of absorbed solution of C40, C50, and C55 changing with the square root of time.

**Figure 5:**
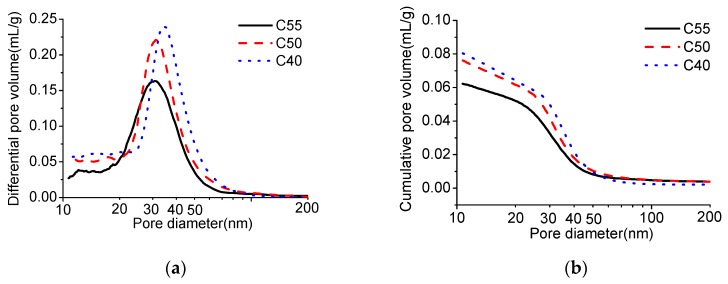
Mercury intrusion porosimetry (MIP) results of C40, C50, and C55. (**a**) Differential pore volume. (**b**) Cumulative pore volume.

**Figure 6:**
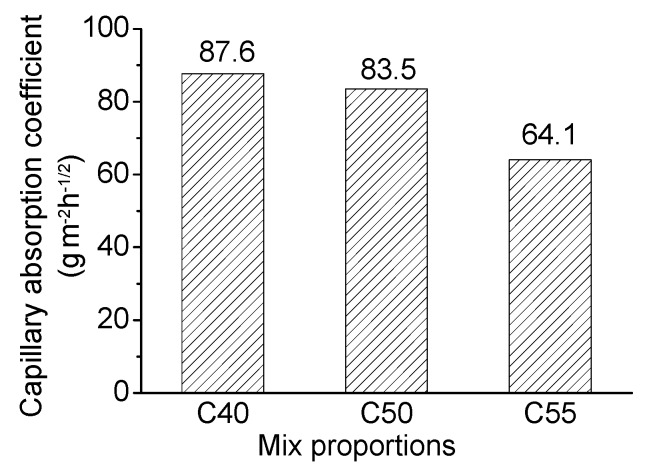
Capillary absorption coefficients of C40, C50, and C55.

Figure 4 shows the relation between the amount of absorbed solution and the square root of time. It can be observed that the amount of absorbed solution increases with time; within the same exposure time, the higher the strength grade of concrete is, the smaller the amount of absorbed solution is. That is because the matrix becomes increasingly denser with the enhancement of strength (see MIP results shown in Figure 5; the total porosity and the most probable pore size of C55 are both lower than that of C40 and C50), which makes it harder for external solution to penetrate under the same saturation degree of concrete. Besides, the capillary absorption coefficients obtained through fitting data in Figure 4 with Equation (2) also decrease with the increase of strength grade, as shown in Figure 6.

### 3.3. Carbonation Resistance

**Figure 7:**
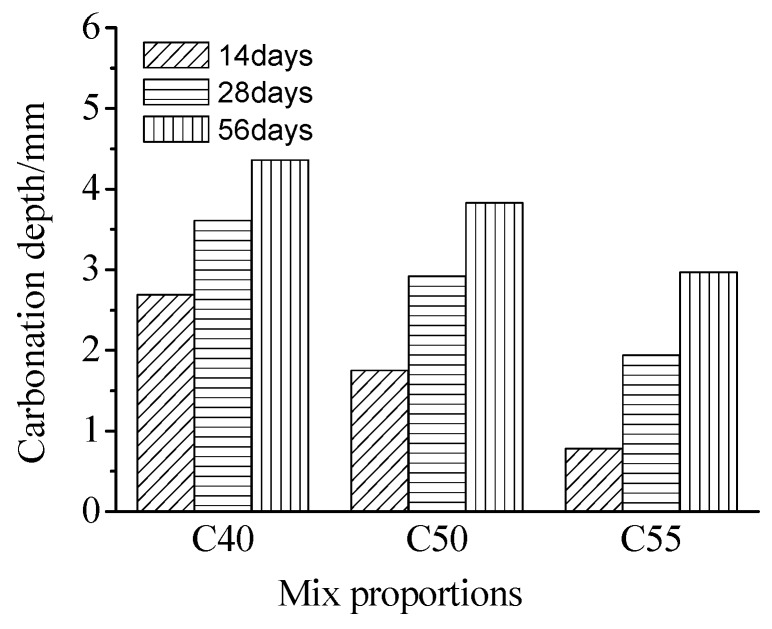
Carbonation depth of C40, C50, and C55 at different exposure times.

Figure 7 presents the carbonation depth of architectural concrete at different exposure times. It can be seen that carbonation depth increases with the carbonation time, which has also been found by numerous studies [29,30,31]. After carbonation for 56 days, the carbonation depth of C40, C50, and C55 are 4.36 mm, 3.83 mm, 2.97 mm, separately, which means that, on the one hand, the carbonation degree decreases with the increase of strength grade, since pore structure of C55 is denser than that of C40 and C50 (see Figure 5). On the other hand, the three mixes are all of a good carbonation resistance, inferring that the carbonation degree will be very low under the atmospheric environment. The results of the studies [25,37] showed that the carbonation resistance of concrete enhanced with the water-to-binder ratio reducing. Several studies [24,25,31,38] found that the carbonation property of concrete was closely related to the compactness of the matrix, and the denser the concrete, the stronger the carbonation resistance. It can be observed that the findings of those investigations are consistent with the findings of this study.

### 3.4. Chloride Penetration Resistance

#### 3.4.1. Atmosphere Zone

(1) Chloride distribution

**Figure 8:**
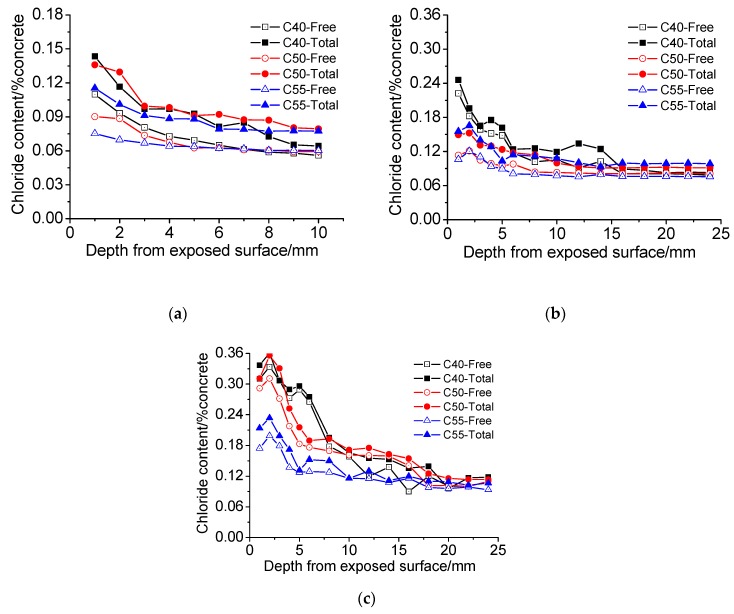
Chloride distribution of C40, C50, and C55 exposed to the atmosphere zone after different times. (**a**) 1 month. (**b**) 3 months. (**c**) 9 months.

The chloride distribution of architectural concrete C40, C50, and C55 exposed to the marine atmosphere zone are presented in Figure 8. It can be seen that the chloride content of all the specimens generally decreases with depth from the exposed surface increasing. While an interesting phenomenon is observed in Figure 8b,c, chloride peaks appear in the surface part of some chloride profiles, which has been also found in numerous studies [39,40,41,42]. According to the research of Ye [43], Joško [44], and Chang [37,45], the effects of both capillary suction–moisture evaporation and carbonation during wetting and drying cycles dominate the formation of chloride peaks. The marine atmosphere zone is also a cyclic wetting–drying environment (rainwater or high humidity air make specimens wet, and sunshine or low humidity air makes them dry). Therefore, with the exposure time increasing, chloride peaks will gradually form in architectural concrete specimens due to the enhancement of capillary the suction–moisture evaporation and carbonation degree.

**Figure 9:**
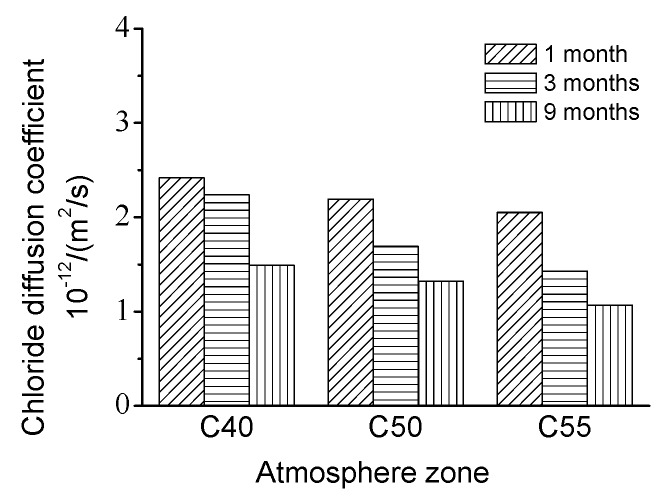
Chloride diffusion coefficient of C40, C50, and C55 exposed to the atmosphere zone after different times.

Moreover, it can also be found that chloride content increases to the decrease of strength grade. Obviously, this is closely related to the pore structures of C40, C50, and C55, as shown in Figure 5. Furthermore, Figure 9 shows the chloride diffusion coefficients (*D*) of these specimens after exposure for different times, which are obtained through fitting the total chloride content with the error function of the basic diffusion equation (see Equation (3)). On the one hand, *D* decreases with the strength grade of specimens, consisting of the change law of matrix density with C40, C50, and C55. On the other hand, all the *D* are quite low, which indicates that chloride penetration resistance of three kinds of architectural concretes is high and this concrete can protect steel bars from chloride attack effectively in the service duration. Besides, the *D* of nine months is lower than those of one month and three months. This is probably because the matrix becomes denser due to continuous hydration after nine months of exposure.

In addition, with the increase of exposure time, both total chloride content and free chloride content increases significantly, and the former of all specimens are always higher than that of the latter.

(3) $$C_{\left(x,t\right)}{=C}_{0}{+(C}_{S}{-C}_{0})\left(1-erf\left[\frac{x}{2\sqrt{Dt}}\right]\right)$$

where *D* is the chloride diffusion coefficient; *C_0_* is the initial chloride content; *C_s_* is the surface chloride content; *t* is the exposure time, *x* is the depth from exposed surface; *C_(x,t)_* is the chloride content at time *t* and depth *x*.

(2) Chloride binding

**Figure 10:**
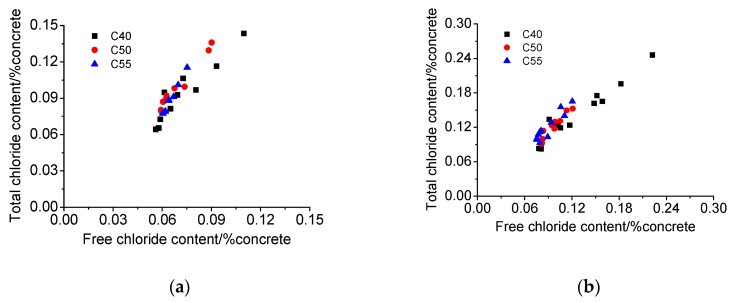

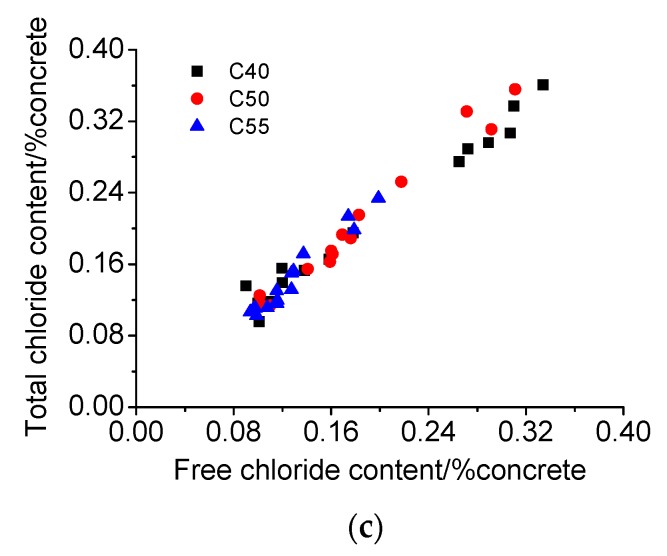
Chloride binding isotherms of C40, C50, and C55 exposed to the atmosphere zone after different times. (**a**) 1 month. (**b**) 3 months. (**c**) 9 months.

Figure 10 shows the chloride binding isotherms of C40, C50, and C55 after exposure for different times. It can be found that there is a positive correlation between total chloride and free chloride. The data distribution of the three mix proportions is relatively concentrated, but it can be observed that, generally, the data distribution of C40 is the lowest, that of C50 in the middle, and that of C55 the highest. The lower the chloride distribution is, the more the free chloride and the fewer the bound chloride. The parameters obtained through the fitting linear function (Equation (4)) are listed in Table 4. It can be seen that the correlated coefficients (R^2^) of all curves are large, indicating that there is a good linear correlation between total chloride and free chloride. Since both total and free chloride decrease with depth, it can be inferred the linear correlation between them also develops with depth, suggesting that the proportion of bound chloride in total chloride at different depths is basically the same. This also means that the architectural concrete of all three mix proportions can resist the heavy CO_2_ penetration, considering that with a large CO_2_ intrusion, different carbonation degrees at different depths of concrete can release bound chloride into the pore solution [46,47], resulting in the increase of proportion of free chloride to total chloride and thus a nonlinear correlation between total chloride and free chloride along with depth.

**Figure 11:**
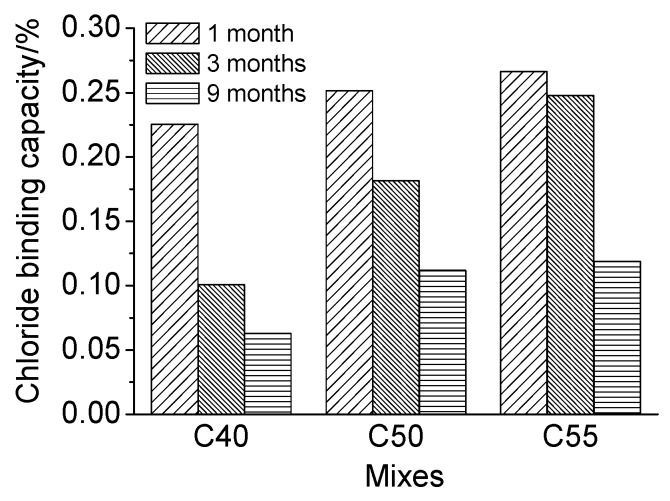
Chloride binding capacity of C40, C50, and C55 exposed to the atmosphere zone after different times.

Moreover, the proportion of bound chloride in total chloride *β* was obtained with parameter *α* and Equation (5) to reflect the chloride binding capacity of concrete of different mix proportions more intuitively. The calculated results are presented in Figure 11. It can be seen that the chloride binding capacity increases with the strength grade of concrete at whatever exposure time. According to the mix proportions of architectural concrete shown in Table 2, concrete of higher strength contains more cement, FA, and SL. The increase of those cementitious-based materials boosts the content of C–S–H gel and AFm [48,49], which are two main hydration products binding chlorides physically and chemically, separately [5,46]. Therefore, the proportion of bound chloride in total chloride of C55 is the highest. The results of Florea, Plusquellec, and Shi et al. [46,47,48,49,50,51] have also presented that bound chlorides increase with the increase of C–S–H gel or AFm content. Moreover, according to the studies [3,46,48,52], C_3_A is deemed an important mineral composition closely related to the formation of bound chlorides in concrete, and the higher the C_3_A content, the more the bound chlorides. The C_3_A content of cement used in this study is about 9.4%, and thus, the chloride binding capacity of C40, C50, and C55 increases gradually, considering that the cement content of C40, C50, and C55 increases successively. Therefore, the enhancement of the chloride binding capacity of the matrix can better protect steel bars inside from corrosion induced by free chloride.

Furthermore, from Figure 11, it can be observed that *β* decreases gradually with exposure time increasing. The increase rate of free chloride is faster than that of bound chloride since free chloride can be immediately formed once external chlorides enter into the pastes, while the formation of bound chloride takes time. Besides, the content of bound chloride in the matrix cannot increase infinitely [46,53] because once the hydration products are saturated from binding, no more bound chlorides can be formed, and the successive penetrated chloride ions can only exist as free chlorides. In consequence, the relative content of bound chloride decreases with exposure time increasing.

*C_t_* = *α*·*C_f_* (4)

where *C_t_* is total chloride content; *C_f_* is free chloride content; *α* is the slope.

*β* = 1 − 1/*α* (5)

where *β* is the proportion of bound chloride content to total chloride content.

#### 3.4.2. Tidal Zone

(1) Chloride distribution

**Figure 12:**
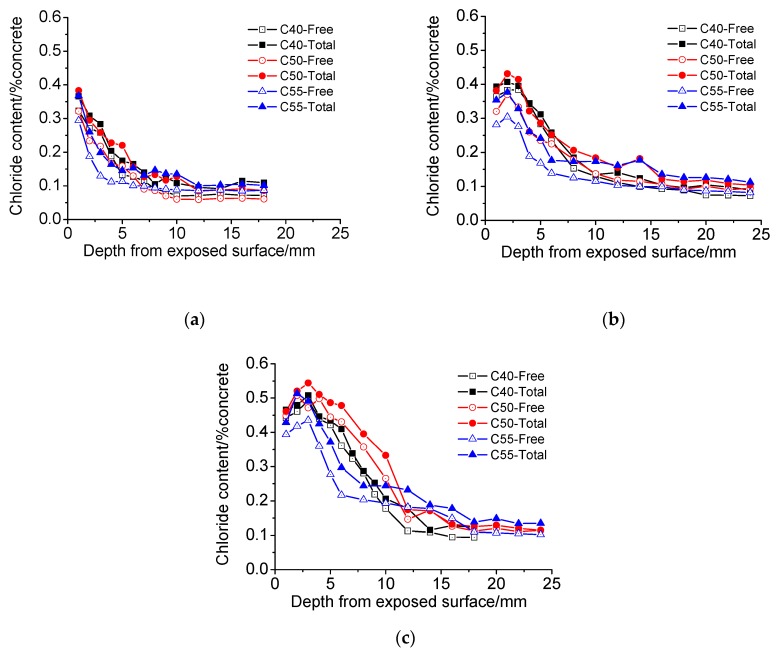
Chloride distribution of C40, C50, and C55 exposed to the tidal zone after different times. (**a**) 1 month. (**b**) 3 months. (**c**) 9 months.

Figure 12 shows the chloride profiles of C40, C50, and C55 exposed to the marine tidal zone. It can be found that the chloride distribution of concrete in the tidal zone is similar to that in the atmosphere zone. The content of total chloride is higher than that of free chloride, and they both generally decrease with the depth increasing; chloride content drops with the strength grade increasing (the penetration of chloride into concrete in the tidal zone is mainly driven by capillary suction, and the higher the strength grade, the smaller the amount of absorbed solution and capillary absorption coefficient, as shown in Figure 4 and Figure 6); chloride content increases significantly with exposure time rising; chloride peaks form in the surface of concrete in the tidal zone, which is a typical cyclic wetting–drying environment. Moreover, the difference is that chloride content in the tidal zone is significantly higher than that in the atmosphere zone, which can be attributed to the mass chloride penetration through direct contact with seawater in the tidal zone. The direct contact with seawater also boosts the capillary suction effect, leading to larger chloride content and depth of peaks in the tidal zone than that in the atmosphere zone [43,44,45].

(2) Chloride binding

**Figure 13:**
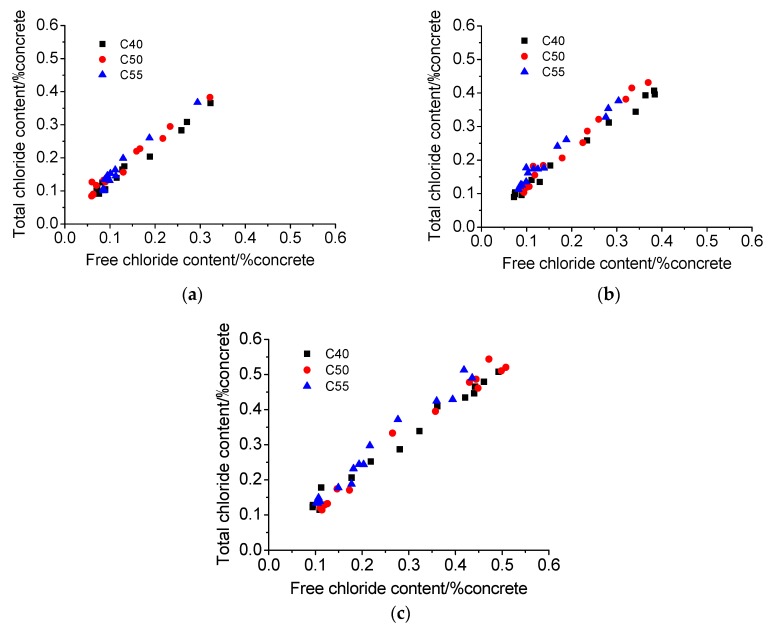
Chloride binding isotherms of C40, C50, and C55 exposed to the tidal zone after different times. (**a**) 1 month. (**b**) 3 months. (**c**) 9 months.

**Figure 14:**
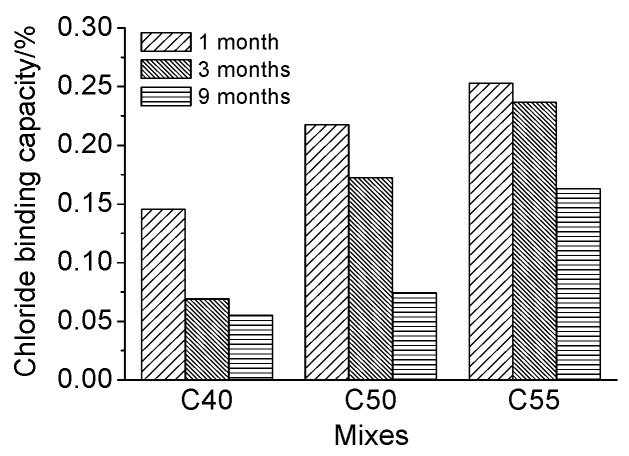
Chloride binding capacity of C40, C50, and C55 exposed to the tidal zone after different times.

Figure 13 presents the quantitative relationship between total chloride and free chloride of specimen subject to the marine tidal zone. It can be observed they both also present a good linear correlation, as suggested by *R*^2^ listed in Table 5. Meanwhile, the proportion of bound chloride in total chloride at different depths is basically the same just like that in the atmosphere zone for limited carbonation effect. Moreover, the *β* obtained through Equations (4) and (5) is presented in Figure 14. It can be observed that, due to more gel materials contained in concretes of a higher strength grade, the relative content of bound chloride in C55 is slightly greater than that in C50 and C40. Besides, the relative content of bound chloride also increases with exposure time.

Furthermore, from Figure 11 and Figure 14, it can be observed that at whatever exposure time and strength grade, the relative content of bound chloride of concrete exposed to the tidal zone is a little lower than that of concrete exposed to the atmosphere zone. It is because that concrete in the tidal zone can directly contact with seawater, and thus more chloride ions can penetrate into the matrix and exist as free chloride. Given that the chloride binding capacity of the matrix is limited by the number of aluminate phases, the *β* of concrete in the tidal zone is lower.

### 3.5. Surface Appearance

#### 3.5.1. Carbonation

**Figure 15:**
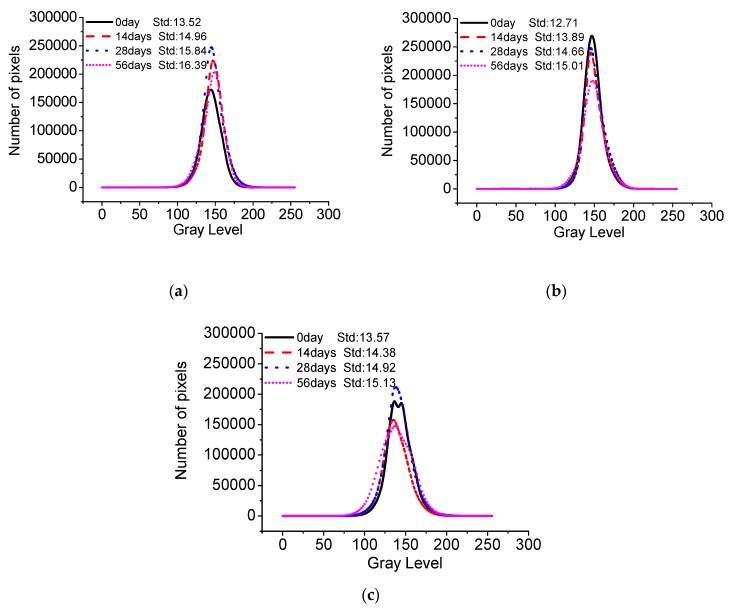
Gray of C40, C50, and C55 exposed to carbonation environment after different exposure times. (**a**) C40. (**b**) C50. (**c**) C55.

**Figure 16:**
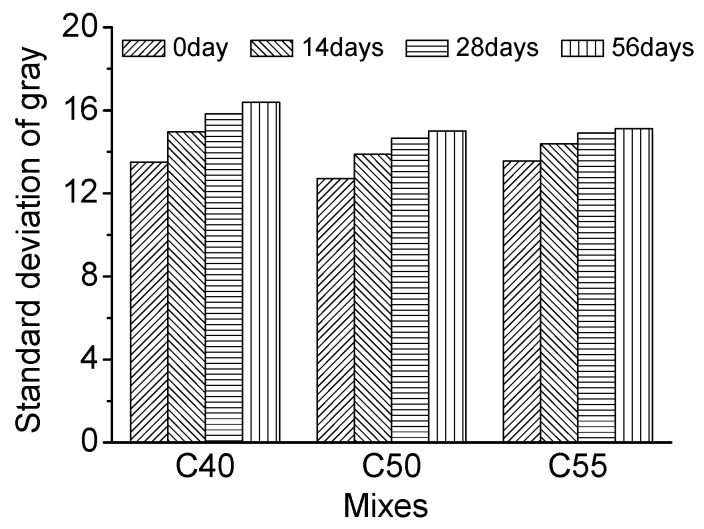
Standard deviation of gray of C40, C50, and C55 exposed to carbonation environment after different exposure times.

Figure 15 shows the gray curves of C40, C50, and C55 after different carbonation times. Using those gray curves and methods introduced in the experiments section, the standard deviation of gray of architectural concrete can be obtained, presented in Figure 16. It can be seen that the standard deviation of gray of three mixtures all increases with carbonation time. The greater the standard deviation is, the worse the surface-color-consistency is, revealing that carbonation is detrimental to the surface quality of architectural concrete. Since concrete material is a heterogeneous material with unevenly distributed pores of various sizes, it can cause the inconsistency of the carbonation effect. As a result, the calcium carbonate crystals generated by carbonation unevenly distribute and accumulate in the surface of concrete, which may be the major factor for the failure of surface-color-consistency. Therefore, carbonation should be given great attention to architectural concrete structures in long service.

**Figure 17:**
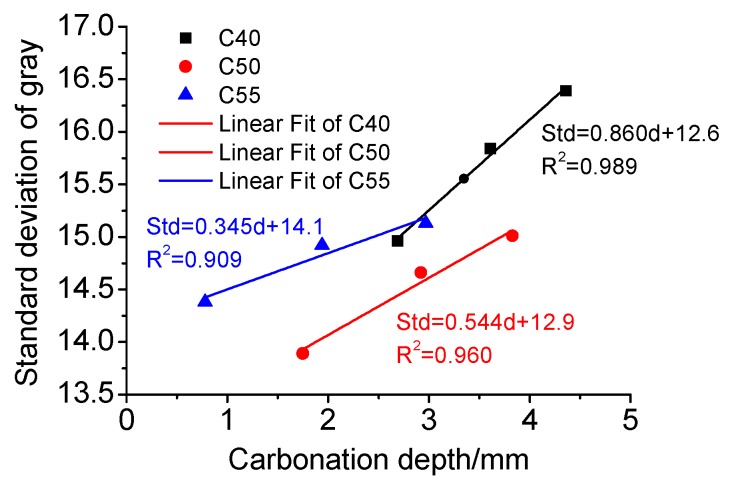
Relationship between standard deviation of gray and carbonation depth.

Furthermore, combining Figure 6 and Figure 16, the relationship between the standard deviation of gray and carbonation depth is presented in Figure 17. It can be found that the standard deviation of gray increases linearly with carbonation depth, indicating that enhanced carbonation is harmful to surface quality. In addition, the slope of the three mixtures decreases with strength grade increasing, suggesting that the higher the strength grade is, the harder it is for carbonation to damage the surface-color-consistency of architectural concrete, which is apparently related to the weaker carbonation effect in concretes of higher strength grade.

#### 3.5.2. Salt Fog

**Figure 18:**
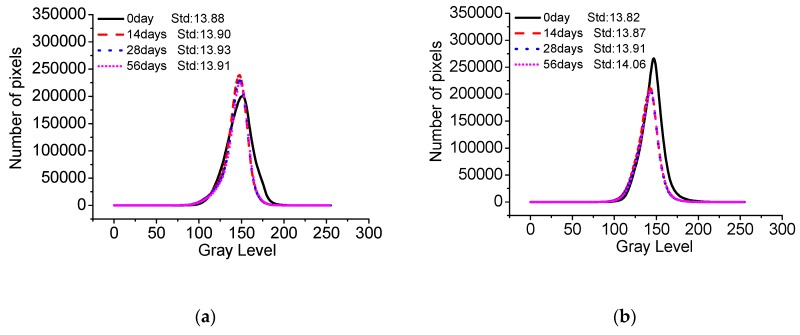

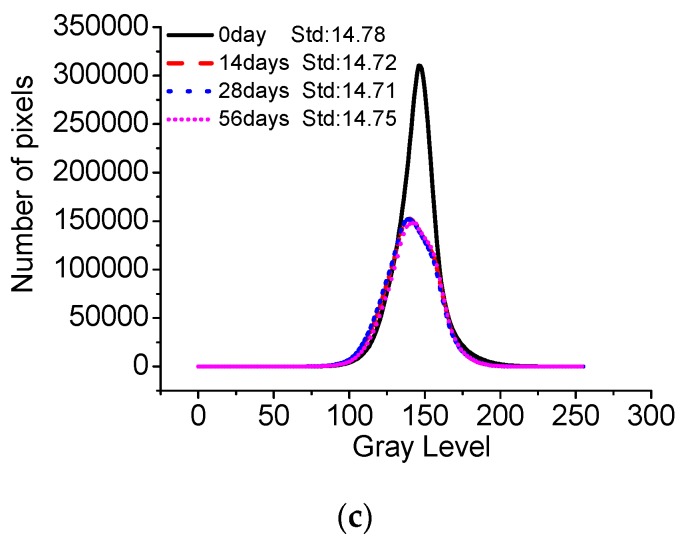
Gray of C40, C50, and C55 exposed to salt fog environment after different exposure times. (**a**) C40. (**b**) C50. (**c**) C55.

**Figure 19:**
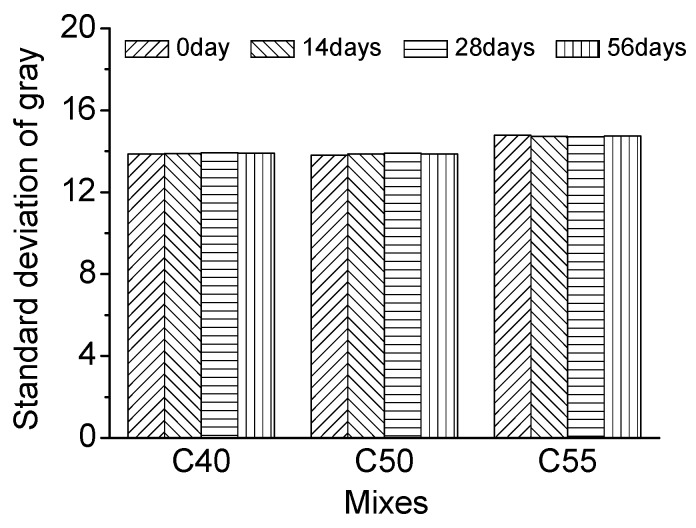
Standard deviation of gray of C40, C50, and C55 exposed to salt fog environment after different exposure times.

Figure 18 shows the gray curves of C40, C50, and C55 under salt fog conditions after different times, and Figure 19 presents the evolution of the standard deviation of gray with exposure time. It can be found that the standard deviation of gray only changes slightly after different exposure times, within a 0.5% range compared with the initial standard deviation of gray. It proves that salt fog will not do severe damage to the surface-color-consistency of architectural concrete.

#### 3.5.3. Atmosphere Zone

**Figure 20:**
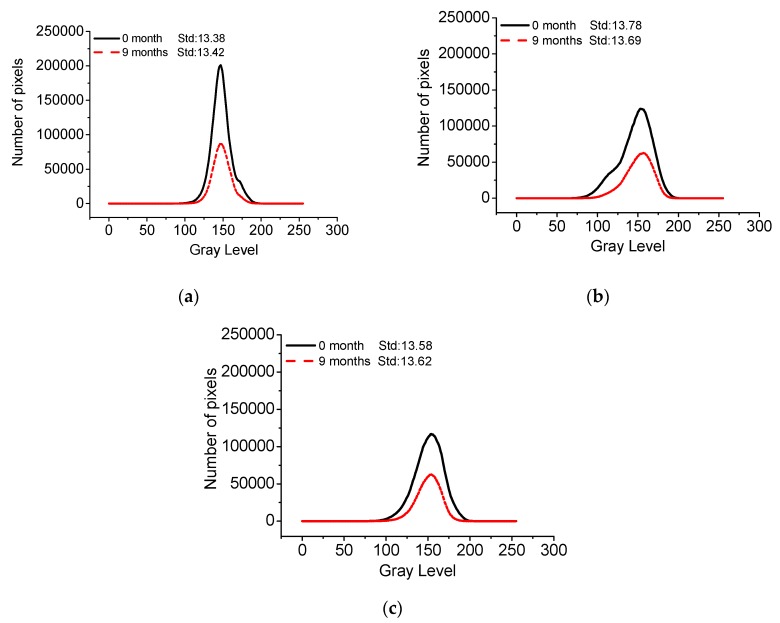
Gray of C40, C50, and C55 exposed to the atmosphere zone after different exposure times. (**a**) C40. (**b**) C50. (**c**) C55.

**Figure 21:**
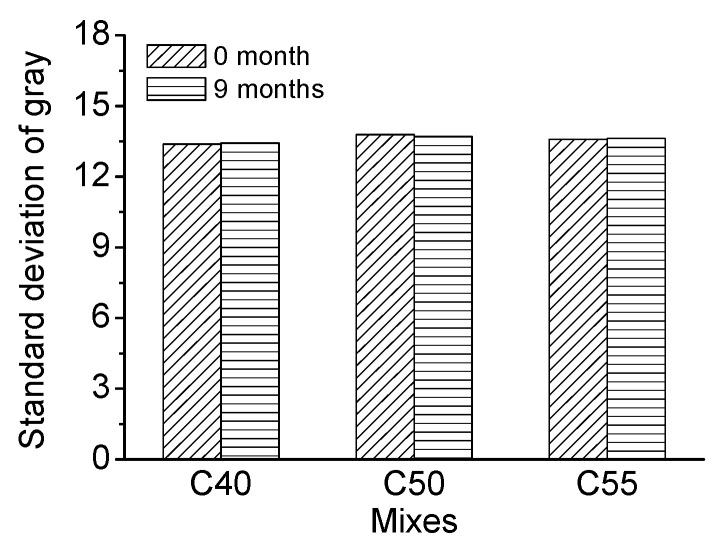
Standard deviation of C40, C50, and C55 exposed to the atmosphere zone after different exposure times.

Figure 20 shows the gray curves of C40, C50, and C55 subject to the atmosphere zone after exposure for nine months, and Figure 21 presents the corresponding standard deviation of gray. It can be found that the standard deviation of gray of three mixtures concrete only changes slightly within the 0.7% range overall, suggesting that chloride in the oceanic atmosphere zone has limited influence on the surface-color-consistency of architectural concrete.

In summary, chloride penetration through the marine atmosphere zone has a very limited impact on the surface-color-consistency of architectural concrete. On the contrary, carbonation has a profound influence on surface-color-consistency. Therefore, the influence of carbonation should be carefully handled to maintain the aesthetic appearance of architectural concrete in long-term service.

## 4. Conclusions

(1) The chloride distributions of architectural concrete in the marine atmosphere zone and tidal zone are similar. The total and free chloride content generally decreases with depth; chloride content augments significantly with exposure time, forming a maximum chloride peak near the surface of concrete. Besides, the chloride diffusion coefficient is small throughout the measurements, indicating that architectural concrete possesses superior capacity on resisting chloride penetration. Moreover, architectural concrete also has great carbonation resistance based on carbonation depth data obtained from the accelerated carbonation experiment.
(2) Concrete of higher strength grade has better chloride binding capacity due to a larger quantity of hydration products and a more densified microstructure, both of which are beneficial for the chloride penetration resistance of concrete.
(3) The standard deviation of surface gray levels of architectural concrete only changes slightly after being exposed to salt fog and the marine atmosphere zone for different times, suggesting that chloride penetration has a very limited impact on the surface-color-consistency. On the contrary, carbonation has a profound influence on the surface-color-consistency; the higher the carbonation degree, the worse the surface-color-consistency. Therefore, the influence of carbonation should be carefully handled to maintain the aesthetic appearance of architectural concrete in long-term service.

## Figures and Tables

**Table 1:** Chemical composition (%) of cement P.I. 52.5, fly ash (FA), and blast-furnace slag (SL).

Binder	CaO	SiO_2_	Al_2_O_3_	MgO	Fe_2_O_3_	SO_3_	K_2_O	TiO_2_	Na_2_O	MnO	Other	LOI
P.I.52.5	52.7	19.9	6.4	4.6	2.8	2.6	0.7	0.4	0.2	0.1	9.6	3.5
FA	8.2	48.8	24.2	1.3	6.5	1.4	2.1	1.3	1.1	/	5.1	1
SL	36.4	29.1	14.3	8.9	0.3	2.0	0.6	1.6	0.3	0.6	5.9	0.37

**Table 2:** Mix proportions of architectural concrete (kg/m^3^).

NO	*w/b*	Cement	SL	FA	Fine Aggregate	Coarse Aggregate	Water	SP
C40	0.33	173	86	86	780	1169	113	4.50
C50	0.30	217	109	109	738	1108	133	5.70
C55	0.27	237	118	118	721	1081	119	6.20

**Table 3:** The slump, extended degree, and compressive strength of architectural concrete.

NO	Slump/mm	Extended Degree/mm	Compressive Strength/MPa		
			3 days	28 days	56 days
C40	185	380	28.9	53.1	56.6
C50	188	410	37.4	64.2	68.0
C55	220	435	44.7	72.0	74.8

**Table 4:** Slope (*α*) and correlated coefficient (*R*^2^) of chloride binding isotherms.

	1 Month		3 Months		9 Months	
	*α*	*R*^2^	*α*	*R*^2^	*α*	*R*^2^
C40	1.291	0.992	1.112	0.993	1.067	0.995
C50	1.336	0.998	1.222	0.995	1.126	0.997
C55	1.363	0.995	1.329	0.995	1.135	0.995

**Table 5:** Slope (*α*) and correlated coefficient (*R*^2^) of chloride binding isotherms.

	1 Month		3 Months		9 Months	
	*α*	*R*^2^	*α*	*R*^2^	*α*	*R*^2^
C40	1.170	0.989	1.074	0.996	1.058	0.995
C50	1.278	0.987	1.208	0.996	1.080	0.996
C55	1.339	0.991	1.310	0.990	1.195	0.994
